# Induction Chemotherapy Followed by Radiation Therapy Versus Surgery Followed by Concurrent Chemo-Radiation Therapy in Locally Advanced Squamous Cell Carcinoma of the Oral Cavity

**DOI:** 10.7759/cureus.15723

**Published:** 2021-06-17

**Authors:** Shabbir Hussain A. Ali, Abdul Hafeez, Ayesha Javed, Mahwish Akhtar, Muhammad Jawaid Mallick

**Affiliations:** 1 Oncology, Ziauddin University, Karachi, PAK; 2 Oncology, Karachi Institute of Radiotherapy and Nuclear Medicine (KIRAN) Hospital, Karachi, PAK; 3 Oncology, Baitul Sukoon Cancer Hospital and Hospice, Karachi, PAK

**Keywords:** concurrent chemo-radiation, radiation therapy, oral cavity cancer, induction chemotherapy, tpf chemotherapy

## Abstract

Introduction

Squamous cell carcinoma of the oral cavity is one of the top 10 malignancies reported globally. Pakistan has a high incidence of oral cancers due to the prevailing poor lifestyle habits/addictions of Pakistanis, and most patients with squamous cell carcinoma present with stage III or IV locally advanced disease. Recommended guidelines indicate surgery as the mainstay of treatment followed by radiotherapy (RT). The addition of induction chemotherapy before surgery or radiation therapy might improve outcomes with increased locoregional control rates.

Methods

This was a retrospective cohort study comparing the outcomes between surgery followed by concurrent chemoradiotherapy (CCRT) and induction chemotherapy followed by RT. This study primarily aimed to evaluate progression-free survival (PFS) and determine the toxicity of chemotherapy.

Results

We found out that the mean PFS among patients undergoing surgery and CCRT and those receiving induction chemotherapy followed by RT were 6.40 (± 2.38) months and 7.6 (± 4.76) months, respectively.

Conclusion

Induction chemotherapy with docetaxel, cisplatin, and 5-fluorouracil followed by RT shows satisfactory results with acceptable toxicity. However, the results are not statistically significant but support the already published data on this treatment aspect of oral cavity cancers.

## Introduction

Squamous cell carcinoma of the oral cavity is the sixth most common neoplasm worldwide. Its incidence varies across different parts of the world. Approximately 405,000 new cases of oral cancer are diagnosed worldwide annually, of which two-thirds are reported in developing countries. The incidence of oral cancer is significantly high in countries such as Pakistan, India, Sri Lanka, and Bangladesh. It accounts for up to 30% of newly diagnosed cases of cancer in developing countries, as opposed to 3% in the United Kingdom and 6% in France, confirming a higher incidence in economically deprived nations [1-2]. It is estimated that in the United States, in 2021, 35,540 new cases were diagnosed with oral cavity cancer, and 6,980 died of the same disease [3].

In developing countries, oral carcinoma is one of the most common cancers, probably due to the risky behaviors practiced in individuals living in these countries [4]. The age-standardized incidence of oral cancers in developing countries is approximately 4.6/100,000 per year. However, the incidence of such cancers is nearly 6.9/100,000 per year in developed countries. Although oral carcinoma has a lower incidence in developing countries, its mortality rate is higher in such low-income countries, at 2.7 per 100,000 compared to 2.3 per 100,000 in developed countries [5]. These statistics are probably due to the lack of incidence rates of oral cancer in South Asian countries where its incidence remains high and due to population differences. However, mortality due to oral cancers is low among men in most countries of Europe and Asia and continues to increase in eastern European countries such as Hungary and Slovakia [5].

Generally, the highest oral cavity cancer rates are found in Melanesia, South-Central Asia, and Central and Eastern Europe, and the lowest oral cavity cancer rates are observed in Africa, Central America, and Eastern Asia for both men and women. Smoking, alcohol use, smokeless tobacco products, and human papillomavirus infections are the major risk factors for oral cavity cancer, with smoking and alcohol having synergistic effects. The contribution of each of these risk factors to the burden varies across regions [6-7]. Worldwide, smoking accounts for 42% of deaths from cancers of the oral cavity (including the pharynx), and heavy alcohol consumption accounts for 16% of the deaths; the corresponding percentages in high-income countries are approximately 70% and 30%, respectively [5]. Smokeless tobacco products and betel quid with or without tobacco are the major risk factors for oral cavity cancer in Taiwan, India, and other neighboring countries [7-8].

Squamous cell carcinoma of the oral cavity is the most common cancer after breast cancer in Pakistan, which is significantly higher than that of other member states of the World Health Organization Eastern Mediterranean regions [9-10]. According to the data published by Jafarey et al. in 1987, it was the second most common cancer in women and the third most common cancer in men [11]. Data published by Burghuri et al. have revealed that between 1995 and 1997, the most common malignancies in Pakistani men were lung cancer followed by oral cancers, and in women, breast cancer ranked one, followed by oral cancers [10]. The age-standardized rates for oral cavity cancers from 1998 to 2002 in Karachi South were 22.5/100,000 in men and 20.4/100,000 in women. Bile et al. [12] have collected data from five leading institutions in Pakistan between 2004 and 2008. Of the 50,552 registered cases, oral cancer was the second leading malignancy (9.9%) after breast cancer (16.1%). Urdu-speaking communities had a higher rate (20.4%), followed by Balochis (19.9%), Sindhis (16.8%), Punjabis (11.7%), and Pushtoons (9.6%) [10]. The major factor associated with oral squamous cell carcinoma is tobacco consumption among Pakistani individuals. Pakistan, as a whole, and especially Karachi, is affected by these smoking habits, which are considered social norms. This is partly due to the socio-economic status, dietary regimen/nutritional status, and lifestyle of the population. Most consumers in Karachi are situated in the so-called *katchi abadis* (which are illegal settlements in the megacity) [13-14].

Approximately one-third of patients with oral cancer present with early-stage disease and these patients are treated with surgery followed by radiotherapy and have a favorable prognosis. Patients with locally advanced tumors of the oral cavity are mainly treated with surgery combined with postoperative chemoradiotherapy. In the last decade, organ preservation protocols using combined chemoradiation (combined systemic treatment with cisplatin and loco-regional radiation) have become the standard of care for locally advanced head and neck cancers. In 2009, the JP Pignon and MACH-NC Collaborative Group presented a meta-analysis of 93 randomized trials with more than 17,000 patients in which the absolute benefit of chemotherapy was 4% in head and neck cancer [15]. Patients with squamous cell carcinoma of the oral cavity who received induction or neoadjuvant chemotherapy with docetaxel, cisplatin, and 5-fluorouracil (5-FU) plus chemoradiotherapy had significantly longer survival than did patients who received cisplatin and fluorouracil induction chemotherapy plus chemoradiotherapy [16]. An Italian Phase II-III trial of 2013 which updated its results in 2017 showed that OS (overall survival) was significantly higher in the induction chemotherapy arm (HR 0.74; 95% CI 0.56-0.97; P = 0.031) [17].

Despite Pakistan being a high-risk population for oral cavity squamous cell carcinomas, there is a lack of randomized studies in the local population to determine the response to induction chemotherapy. Global evidence on the effectiveness of induction chemotherapy is available; thus, induction docetaxel, cisplatin, and 5-FU (TPF) is now becoming an emerging standard in locally advanced oral cancers. This study basically aimed to demonstrate the trends in a single institute regarding locally advanced squamous cell carcinoma of the oral cavity and the comparative response between the two arms after treatment.

This study aimed to compare induction chemotherapy followed by radiotherapy versus surgery followed by concurrent chemoradiotherapy in locally advanced squamous cell carcinomas of the oral cavity in a single institute.

## Materials and methods

This is a retrospective quasi-experimental study conducted in the oncology department of Ziauddin University Hospital. Data of previously treated patients with squamous cell carcinoma of the oral cavity were collected from the medical record department managed between 2010 and 2014 (Total 5 years).

These were further stratified according to our decided inclusion and exclusion criteria. Both men and women aged > 18 years and < 70 years with histologically confirmed, non-metastatic, locally advanced squamous cell carcinoma of the oral cavity (including the upper and lower lips, buccal mucosa, upper and lower alveolus, hard palate, part of the soft palate, anterior tongue, floor of the mouth, and retro-molar trigone) were included in the study. Performance status at the start of treatment was limited to the Eastern Cooperative Oncology Group (ECOG) score of 0 or 1. Patients who were not treated previously with surgery, chemotherapy, or radiation therapy were included in this study. The patients shouldn't have any other simultaneous cancer in the body. Patients who were undergoing any other concurrent anti-cancer treatments were also excluded. Patients' data that were incompletely filled and the patients who lost to follow-up were also excluded.

The total patient source files collected after applying these criteria was 90. The patients were then divided into two arms according to the treatment they were given. Arm 1 included patients who had surgery followed by chemoradiation therapy (66 Gy), concurrent chemotherapy with Cisplatin; these patients came out to be 45. Arm 2 included patients who got induction chemotherapy with TPF protocol (docetaxel 75 mg/m^2 ^Day 1, cisplatin 75 mg/m^2^ Day 1, and 5-FU 1000 mg/m^2^ Days 2-5; 21 days cycle). Two to four cycles were given, after which radiation therapy 66 Gy was given; these were also 45 patients. Data of all the patients were recorded in a questionnaire (see the Appendix). The variables included patients' demographics like age, sex, residential area, native language, substance abuse, and co-morbidity. Other variables include disease factors like the primary site of disease, lymph node involvement, and tumor staging. After completion of treatment, three-monthly follow-up data for 12 months was also recorded.

Outcomes were then recorded, which included progression-free survival (PFS), which is the primary outcome. This was defined as the first recurrence of disease noted clinically and/or radiologically after completion of the given treatment, and it was recorded in months. The other treatment result noted was the toxicity of treatment, and it included neutropenia and oral mucositis. These were recorded in terms of grading for each patient according to the criteria set by the Common Terminology Criteria for Adverse Events (CTCAE) v5.0 developed by the National Cancer Institute (NCI) US in 2017 [18]. These adverse events/toxicity were also stratified according to the number of cycles of chemotherapy given.

Later, after recording all the data, outcomes were then compared in both groups and the difference of mean PFS was found. To check the significance of this difference, the data were analyzed statistically using the independent sample t-test.

## Results

Descriptive results

A total of 90 patients with squamous cell carcinoma of the oral cavity were enrolled in this study as a non-trial cohort, with 45 patients in each arm. The mean age of the patients was 46.73 (± 12.67) years. The mean ages of patients in Arm 1 and Arm 2 were 44.53 (± 11.34) and 48.93 (± 13.65) years, respectively, with no statistically significant difference (p-value, 0.09).

Sex Distribution

**Figure 1:**
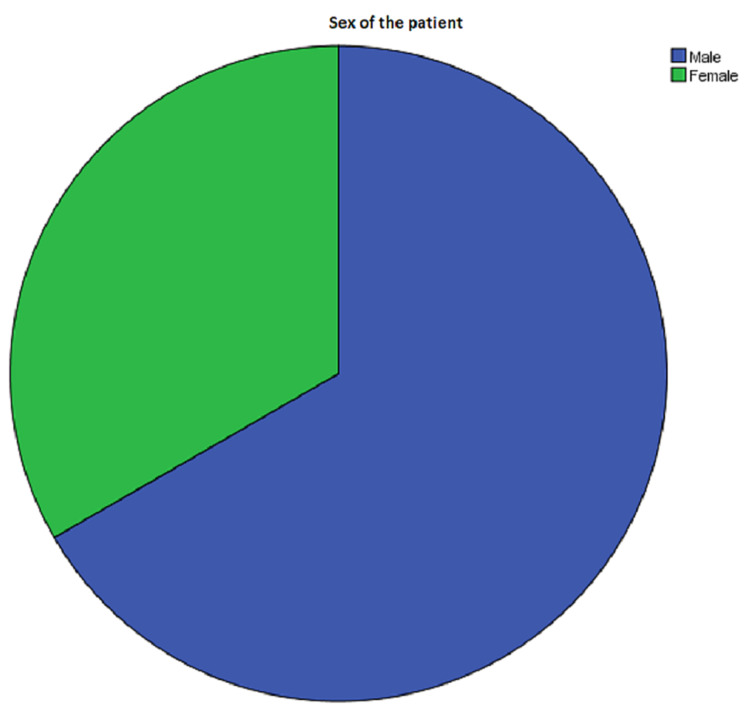
Sex statistics

A total of 60 men and 30 women were included in the trial in both treatment groups (Figure 1).

Residential Area

**Figure 2:**
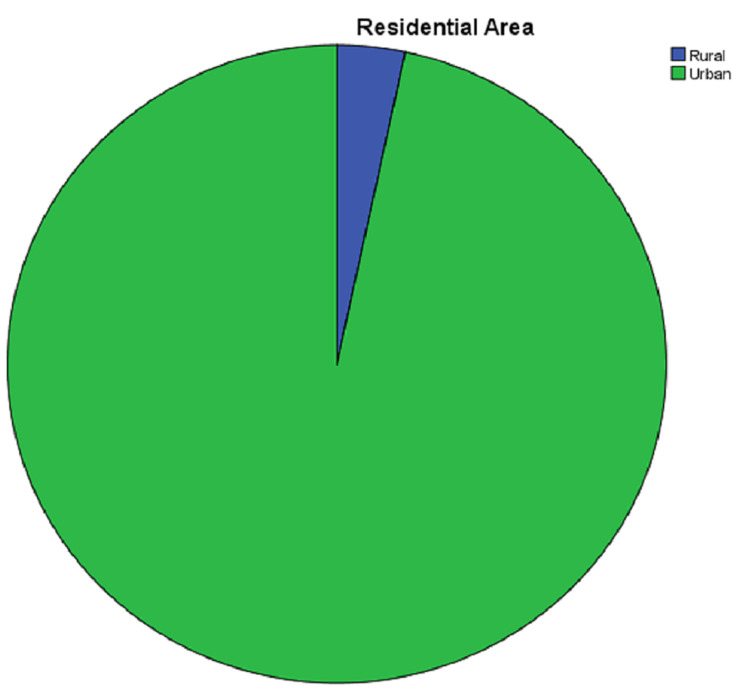
Residential area distribution of patients participating in the study

Among all patients, 87 (96%) were from urban areas, whereas the remaining three lived in rural areas (Figure 2).

Distribution by Native Language

**Figure 3:**
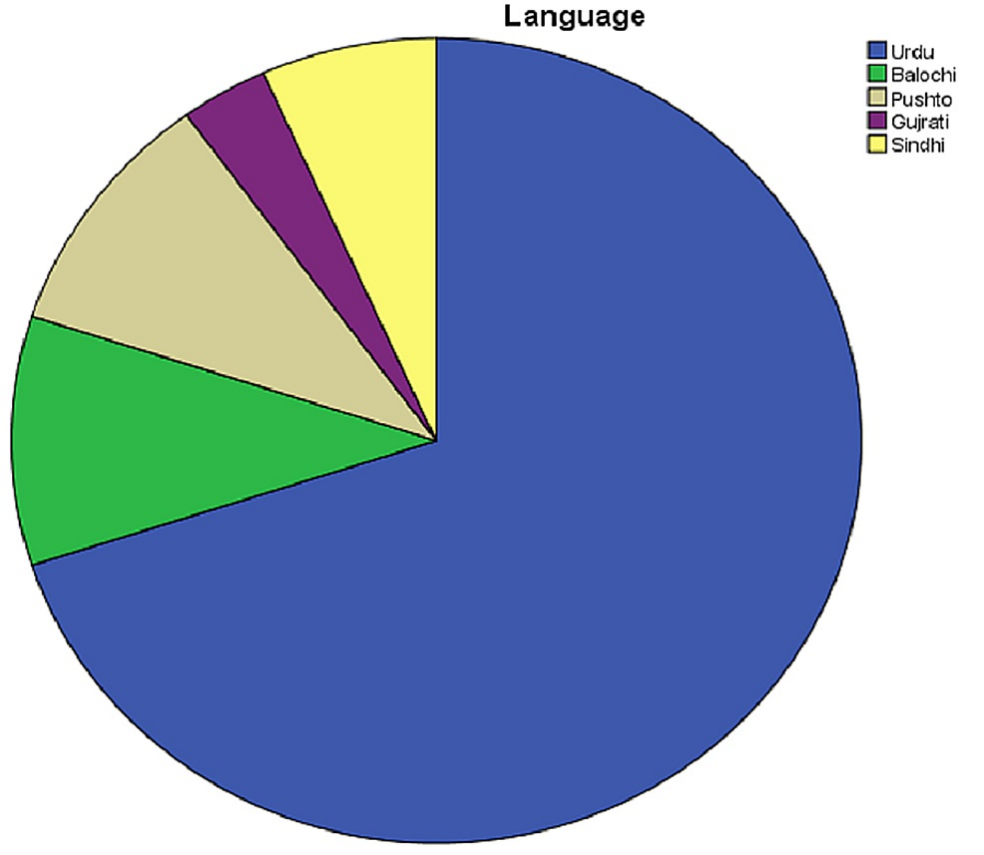
Distribution of patients according to native language

Sixty-three (63; 70%), nine (10%), nine (10%), three (3.33%), and six (6.66%) of the patients were Urdu, Balochi, Pushto, Gujrati, and Sindhi speaking, respectively (Figure 3).

Substance Abuse

**Figure 4:**
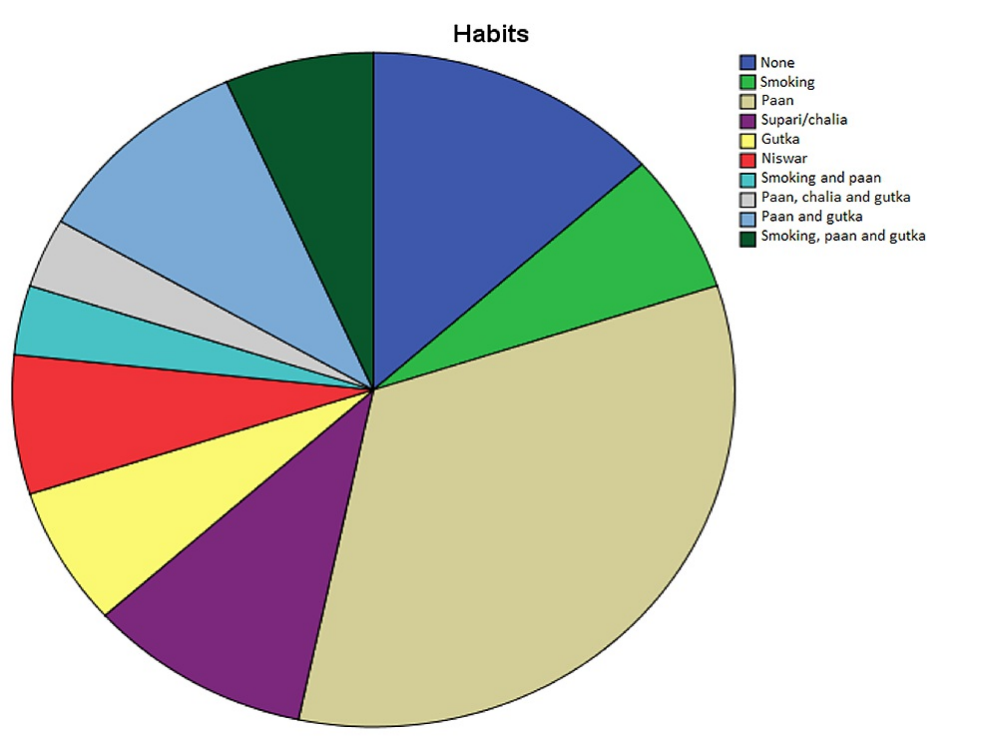
Substance abuse distribution among patients in the study

Only 12 (13.3%) patients were not engaged in any substance abuse, whereas *paan* was the most commonly used substance (56.6%). Other common addictions were smoking (6.66%), *supari/chalia* (10%), *gutka* (6.66%), and *niswar *(6.66%). See Figure 4.

Comorbidities

**Figure 5:**
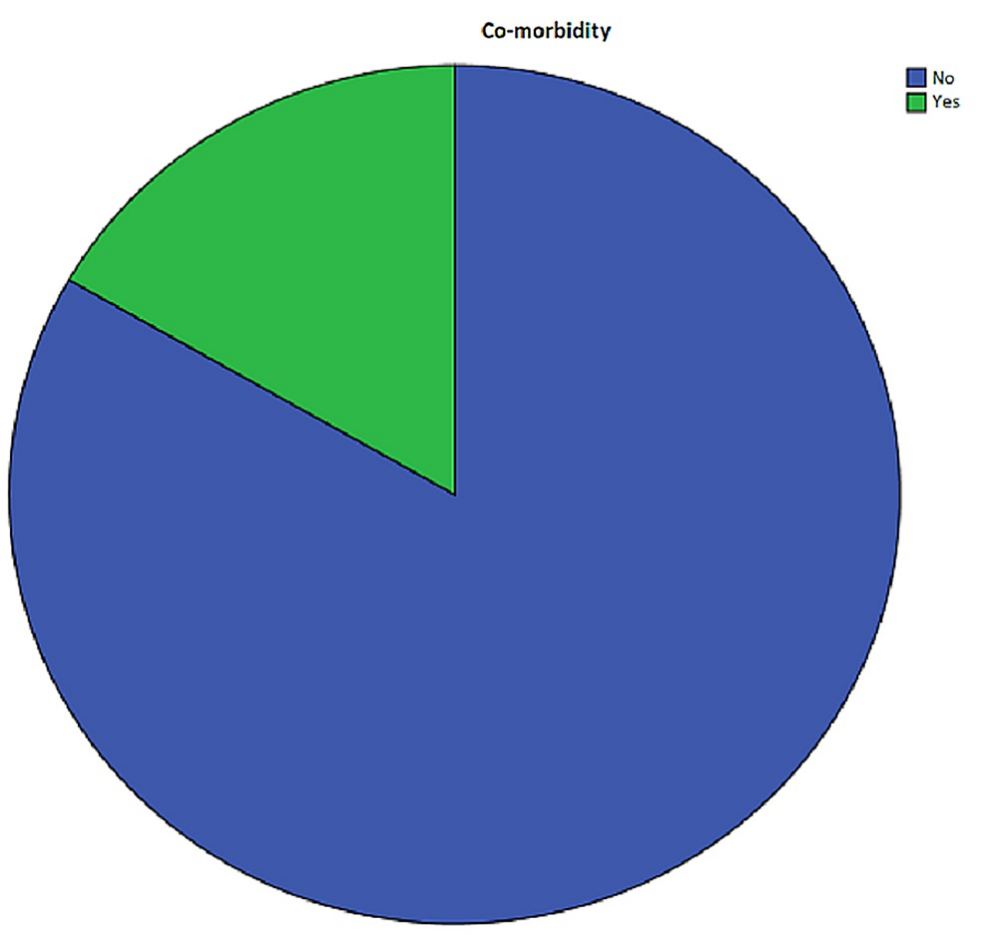
Frequency of comorbidities among patients in the study

Comorbidities (defined as having hypertension, diabetes mellitus, and kidney failure) were present in 15 (16.7%) patients (Figure 5).

Primary Disease Site

**Figure 6:**
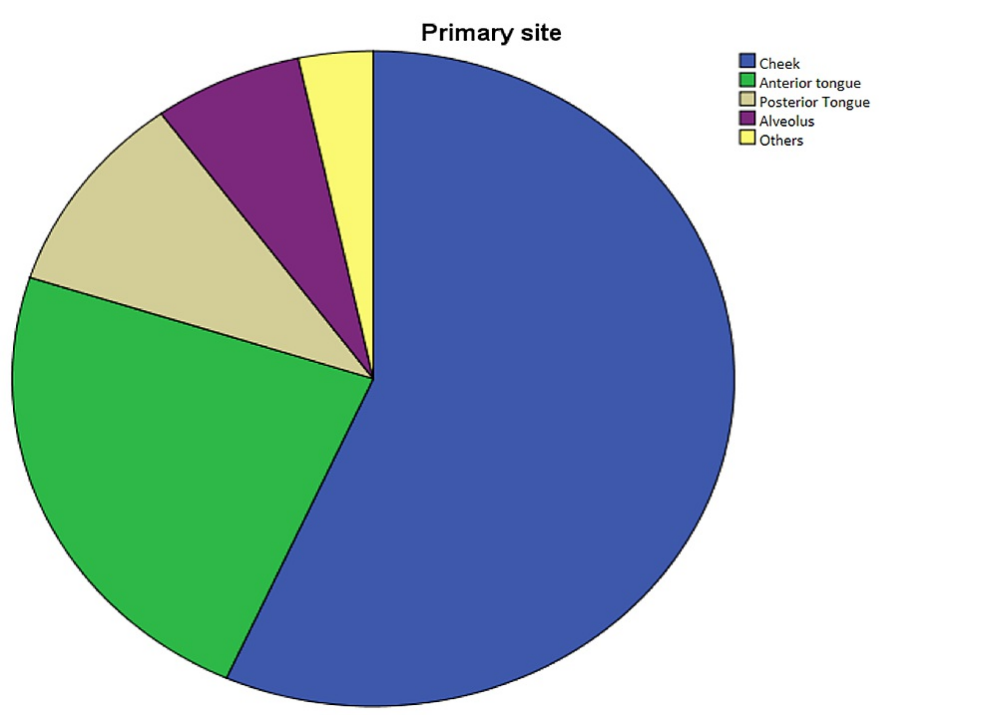
Distribution of primary site of the tumor

Cancers originating in the cheeks were the most common primary site, with 51 (56.7%) patients with this type of tumor. Other sites included anterior tongue (21; 23.33%), posterior tongue (9, 10%), alveolus (6, 6.66%), and others (3, 3.33%). See Figure 6.

Lymph Nodal Involvement

**Figure 7:**
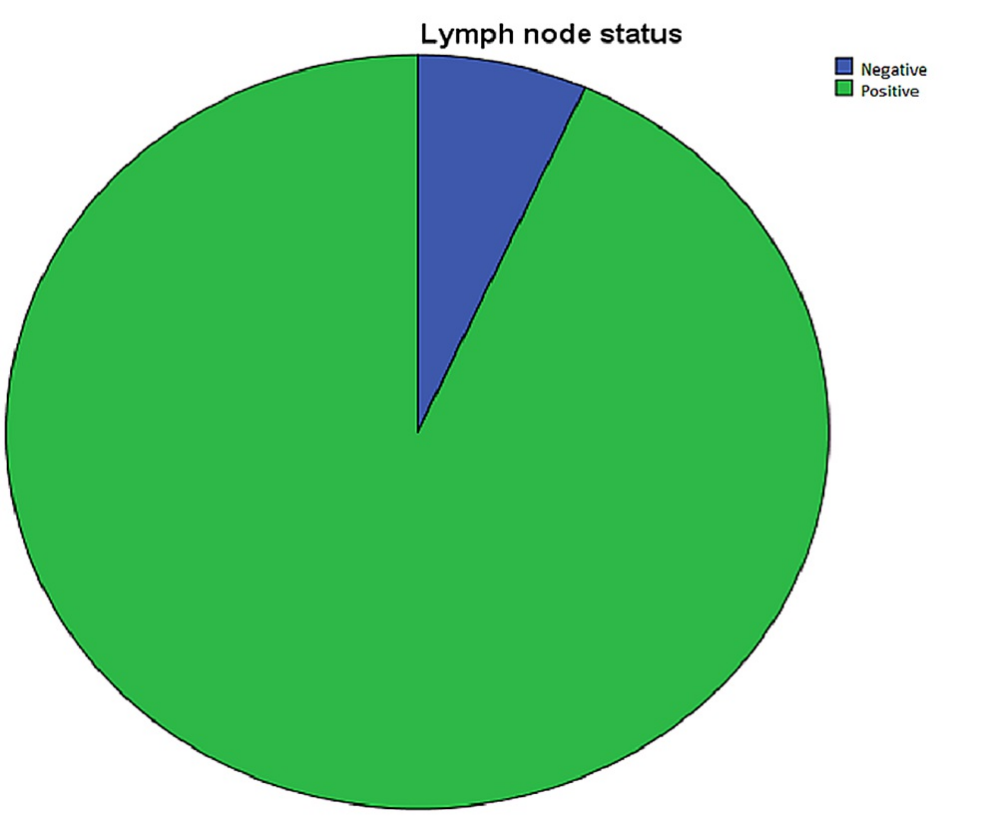
Lymph nodal involvement in patients participating in the study

A total of 84 (93.33%) patients had lymph nodal involvement in both arms (Figure 7).

Tumor Staging

**Figure 8:**
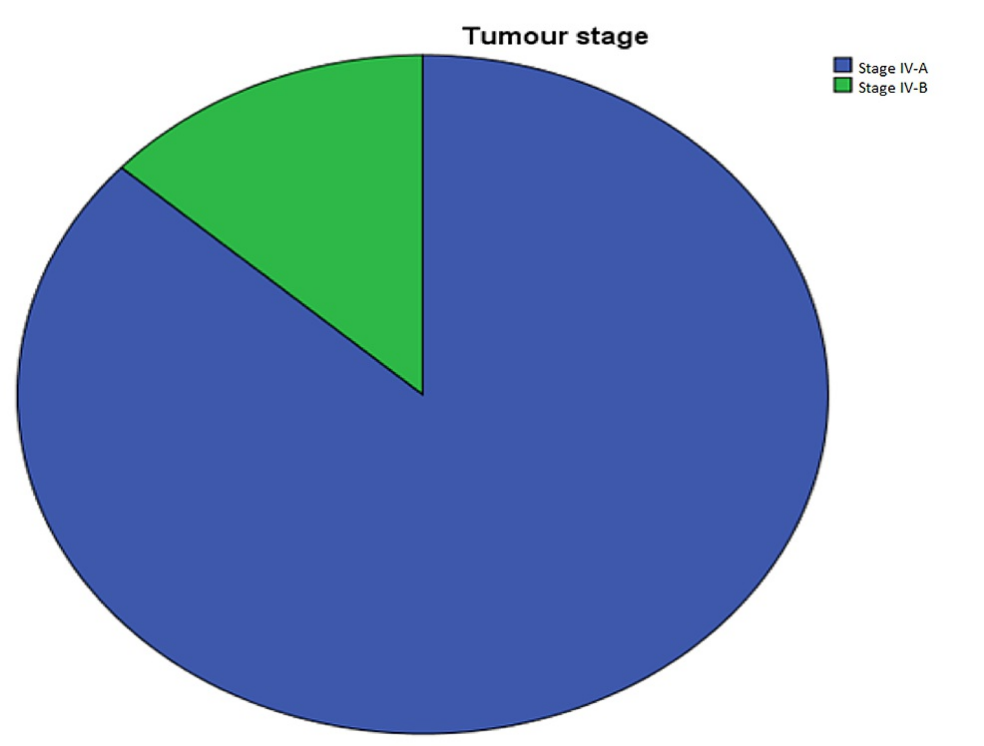
Distribution of tumor-node-metastasis staging in patients participating in the study

In Arm 1, 33 patients were stage IV-A and 12 patients were stage IV-B, whereas, in Arm 2, all 45 patients were stage IV-A (Figure 8).

Inferential results

Toxicity Profile

According to the data, it was evident that patients who received induction chemotherapy had apparent neutropenia and mucositis. The severity of neutropenia was dependent on the number of chemotherapy cycles administered. Furthermore, patients receiving chemoradiation did not develop neutropenia but had oral mucositis. See Table 1.

**Table 1:** Comparisons of neutropenia and oral mucositis in patients of both arms

Adverse events (with number of chemotherapy cycles)	Treatment arm (Frequency and percentage)	
	1	2
Grade of neutropenia		
0	42 (93.33%)	00
1	03 (6.66%)	00
2	00	18 (40%)
3	00	24 (53.33%)
4	00	03 (6.66%)
Grade of oral mucositis		
2	12 (26.66%)	06 (13.33%)
3	15 (33.33%)	12 (26.66%)
4	18 (40%)	27 (60%)
No of cycles of chemotherapy administered		
0	45 (100%)	00
2	00	18 (40%)
3	00	21 (46.66%)
4	00	06 (13.33%)

Mean Progression-Free Survival (PFS)

The mean PFS time of patients undergoing surgery followed by chemoradiation (Arm 1) is shown in Table 2. PFS was calculated according to the patients’ comorbidity, primary disease site, lymph node status, and tumor-node-metastasis (TNM) stage.

**Table 2:** Progression-free survival time in Arm 1

Characteristic	Mean progression-free survival in months
Comorbidity	
Yes	5.00
No	6.00
Primary site	
Lips	--
Cheeks	6.00
Anterior tongue	7.00
Posterior tongue	--
Soft palate	--
Hard palate	--
Alveolus	--
Floor of the mouth	--
Lymph node status	
Positive	6.00
Negative	--
TNM stage	
IV-A	6.00
IV-B	--

The mean PFS of patients undergoing induction chemotherapy followed by radiation therapy (Arm 2) is shown in Table 3. PFS was calculated according to the patients’ comorbidity, primary disease site, lymph node status, and TNM stage.

**Table 3:** Progression-free survival time in Arm 2 TNM: tumor-node-metastasis

Characteristic	Mean progression-free survival in months
Comorbidity	
Yes	8.00
No	7.00
Primary site	
Lips	--
Cheeks	8.00
Anterior tongue	7.00
Posterior tongue	7.00
Soft palate	--
Hard palate	--
Alveolus	10.00
Floor of the mouth	--
Lymph node status	
Positive	8.00
Negative	--
TNM stage	
IV-A	7.00
IV-B	10.00

Based on the results observed in Table 4, when comparing the two arms, there is no significant difference in the PFS between the standard arm and the experimental arm. Therefore, the study concurs with the international guidelines and does not prove any evident benefit of induction chemotherapy; however, a slight difference in favor of the experimental arm is observed, but at the cost of an increased toxicity profile, which concludes that in the upcoming era, we will achieve significant positive results in favor of the induction chemotherapy arm.

**Table 4:** Comparison of progression-free survival times in both treatment arms

	Arm 1 (surgery followed by concurrent chemoradiation therapy [66 Gy], chemotherapy with cisplatin)	Arm 2 (induction chemotherapy [2–4 cycles] with the docetaxel, cisplatin, and 5-fluorouracil protocol, followed by radiation therapy [66 Gy])	p-value
Progression-free survival time in months (mean ± standard deviation)	6.40 ± 2.38	7.60 ± 4.76	0.136

## Discussion

In a country such as Pakistan, patients with head and neck cancers are especially in the unfit group with relatively poor co-morbid conditions, lower socioeconomic status, and dismal educational levels. Considering the prevalence of *paan*, *chalia*, cigarette smoking, *gutka*, and *naswar*, the incidence of oral cancers has significantly increased. Ethnic disparities also exist in our study, as Urdu-speaking Karachiites are the largest consumers of paan and gutka. In our study, a little less than three-quarters of the patients belonged to this ethnic group.

According to previous studies, the most common site for oral squamous cell carcinoma is the buccal mucosa. Similarly, in Pakistan, the majority of cancers develop in the buccal mucosa. According to a study conducted by Yasmin Bhurgri in 2005, a total of 2,253 cases of oral cancer were registered in Karachi South during the eight-year study period (1995-2002). Oral cancers accounted for 8.8% of all cancer cases. Overall, the most common site was the buccal mucosa/cheek (55.9%), followed by the tongue (28.4%), palate (6.8%), gum (4.4%), lip (3.1%), and floor of the mouth (1.4%) [19].

In the present study, the PFS of patients in the experimental arm was higher than that in the standard arm, but the difference between the two groups was non-significant. Moreover, we also recorded lower PFS as compared to the previous literature. which shows the more aggressive nature of squamous cell carcinoma in this region as compared to the Western region. These results are consistent with the trial results reported by Alexander D. Rapidis of Greece [20]. However, in this trial, there was no head-to-head comparison with the standard treatment arm. Furthermore, there are only a few trials comparing the standard of surgery and postoperative radiotherapy and experimental combination of induction chemotherapy followed by radiotherapy alone. In our study, the difference in PFS was not statistically significant between the two groups. This may be due to the smaller number of patients in our study, which might be unable to detect any benefit in the induction chemotherapy arm. Furthermore, shorter follow-up of the patients was also a limitation of our study, which rendered our results statistically insignificant.

Comparing the primary site of the tumor, there was a marginally insignificant difference (p = 0.054) in PFS time in patients with tumors of the cheek. However, comparing the results of PFS in patients with cancers of the anterior tongue, the results were insignificant.

Our study failed to provide any statistically significant evidence, pointing toward a potential role of induction chemotherapy followed by chemoradiotherapy compared to loco-regional treatment alone. This is supported by the results reported by Vokes et al. [21], who demonstrated that docetaxel-and cisplatin-based induction chemotherapy was beneficial in PFS in patients with head and neck cancers. This study, however, has conflicting results, but it still raises the possibility of the potential benefit of induction chemotherapy in oral cancers, which, in some cases, will generate good results if we have a larger-scale, multi-center RCT.

A clinical trial of a similar nature was conducted from February 2014 to October 2015 in three centers of Pakistan (Karachi, Multan, and Faisalabad), named the DECIDE study. The primary objective of this study was to evaluate the overall response rate (including complete response and partial response) of patients treated with combination therapy of docetaxel and cisplatin followed by chemoradiotherapy in patients with locally advanced head and neck cancer. This information was provided through personal communication by the principal investigator of the Karachi Center Professor Dr. S.H.M. Zaidi, which was later published [22]. The results showed a response rate of 68.6% in the induction chemotherapy arm with a manageable safety profile, but they also couldn't report any patient with complete response. This study also supports that induction chemotherapy may help reduce the disease in some way, but it still can't show the best response.

In regard to the limiting factor of the toxicity profile noted in our study, induction chemotherapy may still be tolerable if we apply some measures to reduce the adverse events. The major side effect of neutropenia may be prophylactically treated aggressively with granulocyte-colony stimulating factors (G-CSF); these are given as subcutaneous injections for five days after completion of chemotherapy. Other than this prophylactic antibiotics and care in regards to nutrition has to be taken to counter the immune-compromised state following chemotherapy. Furthermore, intravenous fluids for two to three days can also be supplemented to patients if they are nutritionally depleted. Similarly, oral mucositis may also be treated with antifungals and oral emollients. These measures may definitely help counter the adverse effects of chemotherapy, and we can then opt to give more induction chemotherapy and see if this will help us achieve further benefits in the management of oral cancers.

In our study setting, where resources are limited and the burden of cancers is growing, the majority of patients present with locally advanced disease, especially in head and neck cancers. In such a scenario, where there is an increased risk of local progression of disease due to delayed treatment in terms of delayed or incomplete surgery, induction chemotherapy is a promising modality to be added to the management of oral squamous cell carcinoma. Limiting the tumor after induction chemotherapy (ICT) helps in sparing normal tissue during radiotherapy planning.

Although it remains controversial whether ICT can improve OS, better PFS can assist in achieving a complete response in patients with head and neck cancers [23]. In the later phase, the OS of patients may also improve.

The inability of this study to determine any significant difference in PFS might also be due to the fact that the patients were non-trial cohort patients with comparability issues.

## Conclusions

In this study, induction chemotherapy followed by radiation therapy did not show statistically significant results compared to the standard treatment; however, a slight difference in favor of the experimental arm was observed, but at the cost of increased toxicity profile, which concludes that in the upcoming era, we hope to achieve significant positive results in favor of induction chemotherapy.

## Appendices

**Table 5:** Study questionnaire TNM: tumor-node-metastasis

HEAD AND NECK CANCER QUESTIONNAIRE	
Serial Number	
Name of the patient	
MR number	
Age of the patient	
Sex	
Residential area	
Native language	
Substance abuse	
Co-morbidities	
Primary site of the disease	
Lymph node involvement	(Yes/No)
Group TNM stage	
Neo-adjuvant chemotherapy given	(Yes/No)
Cycles of chemotherapy	
Surgery done	(Yes/No)
Neutropenia during treatment	(Grade 1-4)
Oral mucositis during treatment	(Grade 1-4)
Treatment started on	(Date)
Treatment completed on	(Date)
Recurrence date	
Progression-free survival	(months)
